# Health-economic evaluation of collaborative orthogeriatric care for patients with a hip fracture in Germany: a retrospective cohort study using health and long-term care insurance claims data

**DOI:** 10.1007/s10198-021-01295-z

**Published:** 2021-04-04

**Authors:** Claudia Schulz, Gisela Büchele, Raphael S. Peter, Dietrich Rothenbacher, Christian Brettschneider, Ulrich C. Liener, Clemens Becker, Kilian Rapp, Hans-Helmut König

**Affiliations:** 1 Department of Health Economics and Health Services Research, University Medical Center Hamburg-Eppendorf, Martinistr. 52, 20246 Hamburg, Germany; 2 Institute of Epidemiology and Medical Biometry, Ulm University, Ulm, Germany; 3 Departement of Orthopedic and Trauma Surgery, Marienhospital, Stuttgart, Germany; 4 Department of Clinical Gerontology, Robert-Bosch-Hospital, Stuttgart, Germany

**Keywords:** Orthogeriatric co-management, Economic evaluation, Cost-effectiveness, Entropy balancing

## Abstract

**Background:**

Evidence suggests benefits of orthogeriatric co-management (OGCM) for hip fracture patients. Yet, evidence on cost-effectiveness is limited and based on small datasets. The aim of our study was to conduct an economic evaluation of the German OGCM for geriatric hip fracture patients.

**Methods:**

This retrospective cohort study was based on German health and long-term care insurance data. Individuals were 80 years and older, sustained a hip fracture in 2014, and were treated in hospitals providing OGCM (OGCM group) or standard care (control group). Health care costs from payer and societal perspective, life years gained (LYG) and cost-effectiveness were investigated within 1 year. We applied weighted gamma and two-part models, and entropy balancing to account for the lack of randomisation. We calculated incremental cost-effectiveness ratios (ICER) and employed the net-benefit approach to construct cost-effectiveness acceptability curves.

**Results:**

14,005 patients were treated in OGCM, and 10,512 in standard care hospitals. Total average health care costs per patient were higher in the OGCM group: €1181.53 (*p* < 0.001) from payer perspective, and €1408.21 (*p* < 0.001) from societal perspective. The ICER equalled €52,378.12/ LYG from payer and €75,703.44/ LYG from societal perspective. The probability for cost-effectiveness would be 95% if the willingness-to-pay was higher than €82,000/ LYG from payer, and €95,000/ LYG from societal perspective.

**Conclusion:**

Survival improved in hospitals providing OGCM. Costs were found to increase, driven by inpatient and long-term care. The cost-effectiveness depends on the willingness-to-pay. The ICER is likely to improve with a longer follow-up.

**Supplementary Information:**

The online version contains supplementary material available at 10.1007/s10198-021-01295-z.

## Background

In Germany, there is an ongoing demographic shift in the population. The proportion of older individuals is rising and expected to increase substantially in future decades [1]. In this ageing population, geriatric health conditions will become highly important. Falls are regular events in older and fragile persons [2]. Hip fractures are one of the most severe fall-related injuries which lead to numerous negative health consequences including pain, immobility, reduced quality of life, care dependence and mortality. Additionally, hip fractures cause high health care consumption, as they are expensive in acute and long-term care [3–7]. Both the incidence of hip fractures and the number and severity of negative consequence after hip fracture increase with age [8–10].

For a frail patient of advanced age with several comorbidities, the incident hip fracture may be only one amongst a number of medical issues that often are beyond the scope and expertise of orthopaedic surgeons. To better deal with the special needs of geriatric patients, models for collaborative orthogeriatric care of patients with fragility fractures have been developed in the last years [11–13]. Since then, numerous studies investigated the association of different forms of co-managed, multidisciplinary treatment approaches involving orthopaedic surgeons and geriatricians with health-related outcomes after hip fracture, e.g., mortality and functional status as well as costs.

For in-hospital mortality, systematic reviews found mixed results with partly a statistically significant reduction for patients treated with collaborative orthogeriatric care [12, 14], and partly differences not reaching statistical significance [15–17], compared to standard care. Numerous studies showed statistical significant decreased mortality rates within 30 days [18–24] and 1 year [12, 17, 21, 25, 26], although two meta-analyses found no statistically significant difference in long-term mortality [15, 16].

Another relevant consequence after hip fracture is decreased functional status and, often entailed, care dependence which affects the quality of life and is an important cost driver. Meta-analyses showed that patients treated with collaborative orthogeriatric care regained the same level of activities of daily living (ADL) and their walking ability more often [15] or more often had a return in function after 1 year [14, 17], compared to patients treated with standard care. However, another study did not find differences in mobility and return in function after 1 year [27].

There are several studies with an economic perspective on collaborative orthogeriatric care summarised in a systematic review and meta-analysis [17]. Out of eight studies, seven found a decrease in acute hospital and, if applicable, rehabilitation costs for patients treated with collaborative orthogeriatric care compared to standard care. Only one study found an increase in acute hospital costs [27]. However, after 1-year follow-up this study found a statistically nonsignificant decrease in total costs comprising hospital, rehabilitation, nursing home, and other primary health and care services. A pooled analysis of four studies in the meta-analysis found significantly better health outcomes in terms of reduced 1-year mortality and reduced loss of function after discharge from a hospital or rehabilitation facility. As collaborative orthogeriatric care improved outcomes at lower costs compared to standard care, it was considered economically dominant. Two of the studies additionally found a better quality of life for collaborative orthogeriatric care patients resulting in improved quality-adjusted life years (QALY) [25, 27].

There are different models of collaborative orthogeriatric care investigated in the literature, e.g., treatment in an orthopaedic ward with geriatric consultations on request, or regular, initial treatment in an orthopaedic ward with transfer to a geriatric ward postoperatively, or treatment by an orthopaedic surgeon and a geriatrician together [11, 28]. The literature is still inconclusive as to which of these models is most beneficial. However, models with the early involvement of a geriatric interdisciplinary team seem to be superior compared to others. Accordingly, the precise terms differ in existing literature. In this study, we will henceforth use the term orthogeriatric co-management (OGCM) which in Germany is defined as a hospital treatment by a multidisciplinary geriatric team headed by a geriatrician and delivered either on an orthopaedic or a geriatric ward. A standardised comprehensive geriatric assessment, an early mobilisation and inpatient rehabilitation during the hospital stay starting few days after surgery is provided. In Germany, inpatient rehabilitation measures are usually delivered by either OGCM in a hospital or subacute in a separate facility. However, offering both forms is also possible.

For Germany, there are only a few studies investigating OGCM yet, of which one did not only focus on hip fractures but included further geriatric fractures [29]. Based on a rather small sample, it found statistically insignificant results regarding mortality, ability to walk, complications, as well as costs favouring OGCM. However, it did not investigate cost-effectiveness. Another study with a large dataset [24] found statistically significant results regarding mortality after hip fracture favouring OGCM. Based on the same data as the latter study, we now additionally focused on an economic perspective. The aim of our study was to evaluate associated costs and cost-effectiveness of OGCM in comparison to standard care for hip fracture patients in Germany within 1-year follow-up.

## Data and methods

### Study design, data source, and selection criteria

We used a non-experimental, retrospective, population-based cohort study design. Health and long-term care insurance claims data for the years 2012–2015 were provided by the “Wissenschaftliches Institut der AOK” (“WIdO”). WIdO is a scientific institute and belongs to the largest association of statutory health insurance companies in Germany, which covers about one-third of the German population. Patients were included in our study if they sustained an incident hip fracture (hospital admission diagnosis S72.0 or S72.1 from the 10th revision of the International Classification of Diseases) during the index period of January until December 2014. Starting from the index date of hospital admission, patients were followed for 1 year (follow-up period), or until death. A period of 2 years prior to the index date was stipulated as baseline for determining patient-level risk profiles (see section “Risk adjustment”).

Since we investigated an intervention intended for geriatric patients, a further inclusion criteria was the patients’ age of 80 years or older which corresponds to the definition of geriatric patients by the German Society for Geriatric medicine [30]. Patients who were transferred to another hospital or changed the insurance were excluded. Further exclusion criteria were patients with negative costs due to administrative reasons, and patients treated in hospitals with a low (≤ 80 patients; *n* = 259 hospitals) or ordinary high (> 560 patients; *n* = 10 hospitals) annual hospital volume of hip fracture cases based on AOK data in 2014. The latter was chosen to improve the comparability between intervention and control group and to exclude a possible quality-related source of heterogeneity.

### Intervention

OGCM in German health insurance claims data can be identified by the procedure classification code “OPS8-550”. It identifies a complex treatment of early rehabilitation lasting at least 14 days and provided by a multidisciplinary geriatric team that was headed by a geriatrician and made up of physiotherapists, occupational therapists, specifically trained nurses, social workers, and additional disciplines if needed. The treatment comprises early mobilization after surgery as key component, standardized geriatric assessment, regular interdisciplinary team meetings, and development of a rehabilitation plan with setting of functional goals and a focus on geriatric syndromes. In patients with hip fracture, this multidisciplinary geriatric treatment usually begins within 24 h after surgery and can be delivered at an orthopaedic or a geriatric ward. OGCM is delivered additionally to the standard care of the hip fracture which is most likely appropriate surgery and rehabilitation with no intended contact to a geriatric ward. Further information can be found elsewhere [24]. Since there is considerable financial reimbursement associated with the OPS8-550 code, data are subject to thorough checking and audit and can be considered highly accurate.

The assignment of patients to intervention and control group was done on hospital level. If a hospital treated at least one patient of our study population with the OPS8-550 procedure, it was considered as providing OGCM facilities. Subsequently, all patients treated in this hospital were assigned to the intervention group. Otherwise, when focusing on patients who actually received an OPS8-550, there would be a selection bias. By definition, an OPS8-550 code requires the patient to survive and receive at least 14 days of treatment. However, using survival as part of the group allocation definition would introduce immortal time bias. Identifying which hospitals provided OGCM based on the coding of patients treated in the hospitals overcomes this problem. Although not all hip fracture patients in OGCM hospitals may have actually received the OGCM, they may still benefit from the presence of a multidisciplinary geriatric team. Thus, a patient was assigned to the intervention group, if his index hospital stay due to the incident osteoporotic fracture was in a hospital defined as providing OGCM, and to the control group, if treated in other hospitals.

### Health care costs

In this study, we investigated direct health care costs. Total direct health care costs comprising costs for inpatient (including inpatient rehabilitation) and outpatient treatment, medication, devices/medical appliances, and long-term care were calculated and summed up during the follow-up. Costs were also calculated for all sectors separately. Additionally, inpatient costs and inpatient length of stay in the index hospital (due to the index hip fracture) and rehabilitation facility (if rehabilitation was conducted within 4 weeks after index hospital discharge) were estimated. Costs were reported in 2015 Euro and adjusted for inflation using the Gross Domestic Product price index [31, 32]. We applied a payer perspective on health care costs. All costs were available from health and long-term care insurance claims data, except for long-term care costs.

Costs for long-term care could not be derived directly, but instead, information on care level and nursing home admission were available per quarterly period. In Germany, long-term care recipients were categorized in one of four care levels (0, 1, 2, 3) by the long-term care insurance based on their required assistance in performing ADL and IADL due to disability. The levels were classified depending on daily time needed for care (e.g., care level 1, 2, and 3 requiring basic care such as washing, feeding, or dressing for at least 0.75, 2, and 4 h daily time, respectively) [33], except for care level 0. Care level 0 is intended for individuals with a limited “everyday competence” which means that they are care dependent with special needs for support and supervision instead of medical care. This may particularly concern individuals with a dementia disease, mental disorder or mental disability. The classification is clearly defined and the assessment of severity routinely conducted by a qualified physician or nurse. Therefore, care level is supposed to be a standardized, differentiated and objective indicator of the degree of functional impairment and, consequently, an appropriate and routinely recorded measurement of care dependence. The reimbursement of long-term care was fixed per care level, depending on the arrangement of care (e.g., whether or not a person received care at home or in a nursing home). Therefore, long-term care costs could be estimated based on care level and nursing home information.

The number of quarterly periods per care level was multiplied with the standardized reimbursement per care level for home-delivered and nursing home care, respectively. For care at nursing homes, the reimbursement rate per care level is fixed. For care at home, the amount of reimbursement provided by the long-term care insurance differs depending on who delivers the care. When formal care is delivered by a professional nursing service, the service is reimbursed directly, which means that the patient receives no money but benefits-in-kind via the nursing services (“Pflegesachleistung”). As an alternative, cash benefits may be paid by the long-term care insurance (“Pflegegeld”), which means that the patient receives money for care purposes to compensate for instance informal care delivered by relatives [34]. This amount of money is lower than what is reimbursed to professional nursing services for providing formal care at home. As we could not derive which patient in our population received benefits-in-kind or cash benefits, we used official German statistics data [34] and stratified them by both age groups and sex. As the majority of care recipients in these statistics is presented as receiving a combination of both, we calculated three different scenarios for the reimbursement of care at home: long-term care insurance costs mainly for cash benefits of patients (minimum long-term care costs), long-term care insurance costs mainly for non-cash benefits-in-kind of patients (maximum long-term care costs), and an equal combination of both (average long-term care costs). The main analysis was carried out for the scenario of average long-term care costs, and additional analyses were calculated for the other scenarios.

In Germany, the long-term care insurance pays for a fraction of the long-term care expenses a care recipient actually has. To enable comparisons of the long-term care costs with other countries with differing reimbursement rates, we additionally calculated long-term care costs from a societal perspective. For long-term care in nursing homes, we applied average costs per care level [35]. For long-term care costs at home, we multiplied the minimum daily time for assistance required per care level [33] with the average costs of formal care, which is equivalent to the substitution costs of informal care [35]. As there is no minimum daily time for assistance given for care level 0, we could not calculate costs as we did for the other care levels. We chose to use the reimbursement rate of the long-term care insurance described above to obtain minimum costs for care level 0 from a payer perspective which, however, underestimates the societal costs. Other health care costs formerly mentioned differ only marginally between the payer’s and the societal perspective, because the health insurance reimburses almost all other costs that apply for the hip fracture treatment.

### Effectiveness

We estimated the survived time within 1 year. As information on mortality was available only on a monthly basis, we additionally considered the end date of insurance in the month of death as date of death to derive information on a daily basis.

As an additional approximation of the cost-utility ratio, we adjusted the survival within 1 year for quality of life in terms of utilitites which we derived from another study based on care levels. We assumed that the increase in care level per patient was a consequence of the hip fracture, and that the quality of life decreased with increasing care dependence and care level [36]. Thus, we matched care-level specific utilities to our data to obtain quality-adjusted life years (QALYs). Utilities were based on data from the AgeCoDe-AgeQualiDe study. This was a prospective cohort study which started in the year 2003 and took place in six study centres across Germany. Participants were recruited from the general population by general practitioners if they were at least 75 years, had no dementia and had at least one contact with the GP within the last 12 months. They were interviewed every 1.5 years. Inter alia, care level and quality of life based on EQ-5D-3L [37] were surveyed. We chose the seventh wave of AgeCoDe, because participants were aged at least 85 years and sufficient observation numbers per care level were available. Thus, the participants were geriatric patients, but not necessarily with a hip fracture. Missing values in the study were imputed by employing the “Multiple Imputation using Chained Equations” approach. Further information on the studies can be found elsewhere [38, 39]. The average quality of life per care level was calculated and transformed to care-level specific utilities using the German tariff [40] (displayed in supplementary Table 1).We then matched the utilities to the hip fracture patients based on the care level within 1 year after hip fracture. Specifically, we multiplied the utilities with the average time per care level per patient within 1 year to obtain QALYs.

### Risk adjustment

The risk of mortality or limited functional ability after hip fracture, which would thus affect care dependence and, therefore, health care costs, is correlated to numerous factors on patient and hospital level [41–43]. Unfortunately, hip fracture patients could not randomly be assigned to the intervention and control group. Observational studies like ours may be subject to selection bias and unbalanced baseline characteristics due to a lack of randomisation. To reduce confounding, the reweighting algorithm entropy balancing (EB) [44] was applied to remove inequalities in the independent variables. EB recalibrates the weight of each control individual in such a way that the control group satisfies pre-specified balancing requirements, i.e., equal mean, variance and skewness as in the treatment group. Thereby, the comparability of the control group to the treatment group is maximised regarding the risk adjustment variables. Among all possible combinations of weights that fulfill these balancing requirements, EB choses the combination that deviates as little as possible from uniform weights. The EB weights for the control group $w_{i}$ are chosen by the following approach that minimizes the entropy distance metric

$$\mathop {\min }\limits_{{w_{i} }} H\left( w \right) = { }\mathop \sum \limits_{i} w_{i} \log \left( {w_{i} /q_{i} } \right)\;{\text{with}}\;q_{i} = 1/n_{0} \;{\text{and}}\;n_{0} = {\text{size of the control group}}$$

subject to the following constraints

$$\mathop \sum \limits_{i} w_{i} c_{ri} \left( {X_{i} } \right) = { }m_{r} \;{\text{with}}\;r \in 1, \ldots R$$

$$\mathop \sum \limits_{i} w_{i} = 1$$

$$w_{i} \ge 0\;{\text{for all }}i$$

$c_{ri} \left( {X_{i} } \right) = { }m_{r}$ defines *R* balance constraints imposed on the moments of the covariate distribution of the control group. $m_{r}$ contains the r^th^ order moment of a particular covariate $X_{j}$ from the treatment group. The moment functions are specified for the control group as $c_{ri} \left( {X_{i} } \right) = { }X_{ij}^{r}$.

EB has been shown to be superior to other approaches like propensity score matching or pruning [44–46]. In contrast to propensity score matching, EB is more effective as it achieves higher covariate balance, does not discard individuals and obviates the need for manual propensity score model specification and balance checking, since balance according to the pre-specified balancing requirements is fulfilled by construction [44]. Furthermore, while propensity score methods often decrease balance on some covariates, EB improves balance for all conditioning variables.

There are potential limitations or EB if the balance constraints are inconsistent, or if there are extreme balance constraints which are very far from the control group data. This may occur if the distributions of the covariates of intervention and control control have little overlap. This was avoided by checking the covariate distributions of the intervention and control group, the EB results (see Table 1), and by excluding few potential outlier covariates with less than 50 observations (i.e., the comorbidities HIV, migraines and tuberculosis). The weights obtained from EB were used in the statistical analysis.

**Table 1:** Descriptive statistics of OGCM and control group before and after EB

Variables at index hip fracture date or during baseline (previous 2 years)	OGCM group (*N* = 14,005)		Control group (*N* = 10,512)			
			Before EB		After EB	
Male sex [%]	19.94 (15.96)		19.78 (15.87)		19.93 (15.96)	
Mean age at index hip fracture date [years]	87.09 (20.27)		87.17 (20.99)		87.09 (20.27)	
Mean care dependence during baseline [quarterly periods]						
In care level 0	0.11 (0.47)		0.11 (0.45)		0.11 (0.47)	
In care level 1	2.48 (10.31)		2.49 (10.19)		2.48 (10.31)	
In care level 2	1.29 (6.77)		1.36 (6.95)		1.29 (6.77)	
In care level 3	0.17 (1)		0.20 (1.18)		0.17 (1.00)	
Living in a nursing home	1.38 (7.62)		1.70 (8.92)		1.38 (7.62)	
Occurence of inpatient costs during baseline [%]	65.26 (22.67)		64.28 (22.96)		65.26 (22.67)	
Mean amount of inpatient costs during baseline [€]	6307.75 (80,949,564)		5660.19 (69,449,468)		6307.18 (80,938,564)	
Mean amount of outpatient costs during baseline [€]	1699.91 (1,413,582.4)		1767.44 (1,436,238)		1700.05 (1,413,687)	
Mean amount of medication costs during baseline [€]	2327.17 (5,613,924)		2335.30 (5,602,612)		2327.21 (5,613,808)	
Mean amount of devices/medical appliances costs during baseline [€]	244.51 (425,981.53)		259.29 (462,341)		244.55 (426,082)	
Mean index hospital volume (annual hip fracture cases)	89.40 (1393.45)		72.05 (1196.98)		89.35 (1,394.46)	
Medication-based comorbidities [%]						
Acid related disorders	51.65 (24.97)		52.20 (24.95)		51.65 (24.98)	
Bone diseases (osteoporosis)	9.41 (8.53)		8.87 (8.08)		9.41 (8.53)	
Cancer	0.56 (0.55)		0.68 (0.67)		0.56 (0.55)	
Cardiovascular diseases (incl. hypertension)	87.43 (10.99)		87.88 (10.65)		87.43 (10.99)	
Dementia	9.93 (8.94)		11.00 (9.79)		9.93 (8.94)	
Diabetes mellitus	21.26 (16.74)		21.01 (16.6)		21.26 (16.74)	
Epilepsy	9.95 (8.96)		9.67 (8.74)		9.95 (8.96)	
Glaucoma	10.02 (9.02)		9.49 (8.59)		10.02 (9.02)	
Gout, Hyperuricemia	13.71 (11.83)		13.35 (11.57)		13.71 (11.83)	
HIV	Excluded due to less than 50 observations					
Hyperlipidemia	28.35 (20.32)		27.94 (20.14)		28.35 (20.32)	
Intestinal inflammatory diseases	0.93 (0.92)		1.14 (1.13)		0.93 (0.92)	
Iron deficiency anemia	8.24 (7.56)		8.53 (7.81)		8.24 (7.56)	
Migraines	Excluded due to less than 50 observations					
Pain	60.50 (23.90)		59.65 (24.07)		60.50 (23.90)	
Parkinson’s disease	7.44 (6.89)		7.35 (6.81)		7.44 (6.89)	
Psycholgical disorders (sleep disorder, depression)	38.46 (23.67)		39.03 (23.8)		38.47 (23.67)	
Psychoses	23.11 (17.77)		25.95 (19.22)		23.11 (17.77)	
Respiratory illness (asthma, COPD)	14.99 (12.74)		15.03 (12.77)		14.99 (12.74)	
Rheumatologic conditions	43.81 (24.62)		43.31 (24.56)		43.81 (24.62)	
Thyroid disorders	21.46 (16.86)		21.48 (16.87)		21.46 (16.86)	
Tuberculosis	Excluded due to less than 50 observations					

Variance is stated in parentheses as it was used for the entropy balancing reweighting algorithm

OGCM = Orthogeriatric co-management; EB = Entropy balancing

The following covariates recorded during the baseline period or at the index hip fracture date were used for EB: Sex; age at the index hospital admission; comorbidities according to the medication during the last 2 years prior to the fracture [47, 48] (except for HIV, migraines and tuberculosis as there were less than 50 observations); number of quarterly periods being care dependent due to care level 0, 1, 2, or 3, respectively, during baseline; number of quarterly periods living in a nursing home during baseline; hospital volume (defined as mean number of hip fracture cases per year in the AOK dataset, weighted with the market share of the AOK per German federal state to avoid bias); and direct health care costs summed up during baseline for inpatient treatment, outpatient treatment, medication, and devices/medical appliances. As a considerable share of the patient had no inpatient costs during baseline, we additionally used the information whether or not inpatient costs per patient occurred at all. To avoid bias by extreme outliers, all costs were winsorised at the 99% percentile.

### Statistical analysis

Health care costs were estimated in two steps. First, not for all patients costs may have occurred (e.g., if they died on the first day of observation or did not utilize outpatient or medication services during follow-up). Therefore, a logistic regression estimated the likelihood for the occurrence of any costs at all. Second, the amount of costs is usually not normally distributed, but always ≥ 0, left skewed and with few extreme outliers. Therefore, we applied a generalised linear model with a gamma distribution and a log link function to estimate the mean amount of costs, if occurred [49]. To simultaneously estimate the occurrence and the amount of costs, a weighted two-part model was applied [50]. As an exception, inpatient costs, as well as inpatient length of stay, occurred for every patient, because the follow-up started at the time of hospital admission. Therefore, both were estimated with a weighted generalised linear model with a gamma and a log link function only. For long-term care costs, weighted mean costs over all patients were calculated and tested between OGCM and control group using *t* tests. Care dependence was estimated in terms of the number of quarterly periods with each care level or living in a nursing home during follow-up. Thus, care dependence was a count variable and, therefore, estimated using a weighted generalised linear model with a Poisson distribution and a log-link function. Life years and QALYs gained were derived with weighted means and tested using *t* tests.

To analyse the cost-effectiveness of the intervention at the end of the follow-up, the incremental cost-effectiveness ratio (ICER) was calculated as follows [51]:

$$ \begin{gathered} {\text{ICER}} = \frac{({\text{Weighted mean costs}}_{{\text{OGCM group}}} {-}{\text{Weighted mean costs}}_{{\text{control group}}} )} {( {{\text{Weighted mean effects}}_{{\text{OGCM group}}} {-}{\text{Weighted mean effects}}_{{\text{control group}}} )} } = \frac{\Delta \overline{{\text{Weighted costs}}}} {\Delta \overline{{\text{Weighted effects}}}} . \end{gathered} $$

Effects were measured as average life years survived within 1-year follow-up, and, additionally, as quality-adjusted years survived within 1-year follow-up. Thus, the ICER informs about the additional costs caused by the intervention to achieve an additional life year compared to standard care. In case the intervention is less costly and more effective, the intervention is cost-effective and said to dominate standard care. However, if the intervention is more costly and more effective, the cost-effectiveness depends on the maximum willingness to pay (WTP) per unit of life years gained.

Next, cost-effectiveness acceptability curves based on the net-benefit approach were constructed to handle uncertainty of the ICER [51, 52]. The net benefit approach reformulates the ICER into a net-monetary benefit (NMB) and considers different maximum WTP. For each WTP, the intervention is cost-effective, if the point estimate of the NMB is positive. The NMB was calculated as follows: ${\text{NMB}} = {\text{WTP}} \times {\Delta }\overline{{{\text{Effects}}}} - {\Delta }\overline{{{\text{Costs}}}}$. Since the WTP was unknown, the WTP was iterated from €0 to a meaningful threshold (in this study €250,000) in steps of €1000. Subsequently, the iterated NMB was used as dependent variable in a weighted regression model with the intervention as independent variable.

The results were presented in a cost-effectiveness acceptability curve (CEAC) which informs about the intervention's probability of being cost-effective at different WTPs. The respective probability is calculated as 1 − *p*/2 if the intervention coefficient of the weighted linear regression model was positive and *p*/2 if the intervention coefficient was negative. P represents the *p* value of the intervention coefficient. We considered the intervention cost-effective if the probability of being cost-effective was above a probability threshold of 95%.

The analyses were repeated for different calculations of long-term care costs (minimum/maximum long-term care costs). All calculations were performed using SAS software v9.4 (SAS Institute Inc, Cary, NC) and Stata 15 (StataCorp, College Station, TX). Because this study comprised analysis of anonymized routine data, it was not necessary to request approval from the ethics committee of the University of Ulm or informed consent from the study participants.

## Results

In total, 24,517 hip fracture patients were investigated, out of which 14,005 were treated in OGCM hospitals and 10,512 in other hospitals. Descriptive characteristics and results from EB are displayed in Table 1. About one-fifth of all patients was male and mean age was 87 years. Within the 2-year baseline period, patients were care dependent according to one of the care levels for about four quarterly periods on average. Intervention and control group mainly differed in the number of quarterly periods patients had been living in nursing homes (higher for control group), in the amount of inpatient costs (higher for OGCM group), and in index hospital volume (higher for OGCM group). However, after EB both groups were virtually equal in terms of mean, variance and skewness of the risk adjustment variables, as displayed in Table 1 and supplementary Table 2.

During follow-up, patients in the intervention group had slightly more quarterly periods with an increased care level, compared to baseline, as reported in supplementary Table 3. However, note that the mean time patients survived (life years) also was higher in the intervention group, compared to the control group.

Estimated mean costs are reported in Table 2 and increased for all categories in the intervention group. Cost difference was €1181.53 (*p* < 0.001) for total costs from a payer perspective and €1408.21 (p < 0.001) from a societal perspective, €1007.25 for inpatient treatment (*p* < 0.001), €1091.18 for inpatient treatment due to the index hip fracture (*p* < 0.001), €29.01 (*p* < 0.01) for outpatient treatment, €38.22 for medications (*p* < 0.05), and €1.38 for devices/medical appliances. Mean costs for long term care resulted in a difference of €109.25 from a payer perspective, and €335.90 (*p* < 0.05) from a societal perspective. The inpatient length of stay due to the index hip fracture was 2.33 days longer in the intervention than in the control group (*p* < 0.001). Due to the lower mortality in the OGCM group, patients survived 0.77 life years during follow-up in the OGCM group, which was 0.02 life years more than in the control group (*p* < 0.001).

**Table 2:** Costs and outcomes during follow-up for OGCM and control group with entropy balancing weights

	OGCM group (*N* = 14,005)	Control group (*N* = 10,512)	Difference	(SE)
Costs [€]				
Total (payer perspective)^a, b^	22,255	21,073	1.181.53***	(154.55)
Total (societal perspective)^a, b^	29,203	27,794	1408.21***	(214.72)
Inpatient^b, c^	13,509	12,505	1007.25***	(110.38)
- Thereof due to index hip fracture^b, c^	10,001	8767	1091.18***	(54.44)
Outpatient^d^	814	785	29.01***	(9.36)
Medication^d^	1096	1057	38.22*	(17.58)
Devices/medical appliances^d^	276	275	1.38	(5.71)
Long-term care (payer perspective)^e^	6561	6452	109.25	(87.14)
Long-term care (societal perspective)^e^	13,508	13,172	335.90*	(162.61)
Inpatient length of stay due to index hip fracture [days]^b, c^	25.97	23.65	2.32***	(0.21)
Life years^e^	0.77	0.74	0.02***	(0.01)
ICER [€ per life year gained]				
from a payer perspective	52,378.12			
from a societal perspective	62,418.54			

*OGCM* Orthogeriatric co-management

*p < 0.05; **p < 0.01; ***p < .001

^a^Total costs comprised inpatient, outpatient, medication, medical aid and long-term care costs

^b^Estimated using a gamma GLM model and entropy balancing weights

^c^Inpatient treatment comprises hospital and inpatient rehabilitation treatment

^d^Estimated using a two-part logistic and gamma model and entropy balancing weights

^e^Weighted mean values. Difference was tested using *t*-test

When repeating the analyses for different scenarios of long-term care costs from a payer perspective, there were only slight differences in total costs (difference for minimum long-term care cost calculation: €1134.84 (*p* < 0.001); difference for maximum long-term care cost calculation: €1197.09 (*p* < 0.001)) and long-term care costs (difference for minimum long-term care cost calculation: €62.50; difference for maximum long-term care cost calculation: €124.83).

**Fig. 1:**
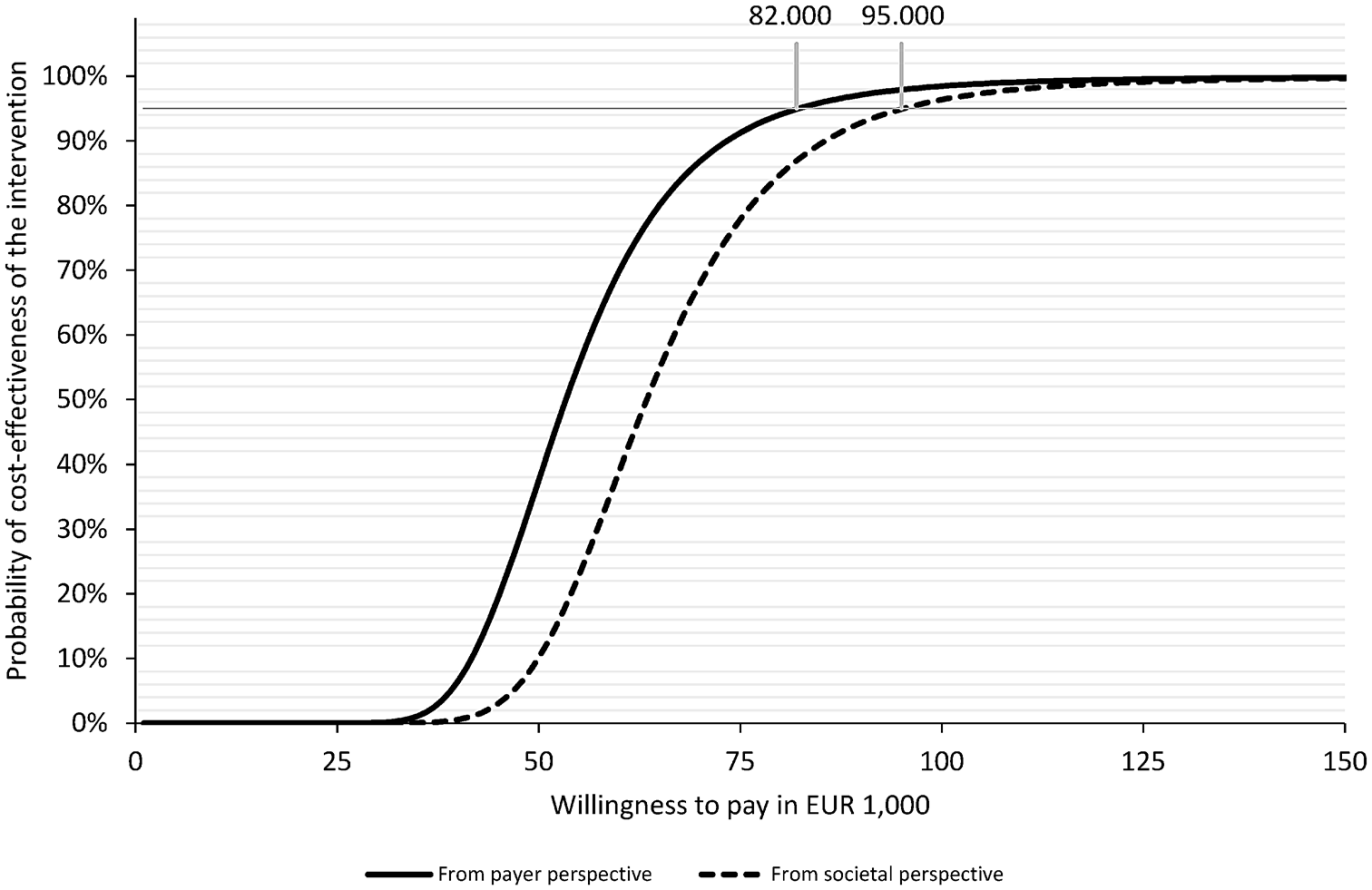
Cost-effectiveness acceptability curve from payer and societal perspective

The ICER based on total costs and life years gained equalled €52,378.12 per life year gained from a payer perspective and €62,418.54 per life year gained from a societal perspective (Table 2). The CEACs are displayed in Fig. 1 and represent the probability of the intervention being cost-effective at different WTPs. If the society was willing to pay at least €82,000 per life year gained, the intervention had a more than 95% probability of being cost-effective from a payer perspective. From a societal perspective, the WTP should be at least €95,000 per life year gained.

Based on the cost–utility approximation, patients on average had 0.51 QALYs during follow-up in the OGCM group, which was 0.02 QALYs more than in the control group (0.49; *p* < 0.001). The ICER based on total costs and QALYs equalled €60,902 per QALY from a payer perspective and €81,614 per QALY from a societal perspective.

## Discussion

In this retrospective cohort study, we evaluated associated costs and cost-effectiveness of hospitals providing collaborative orthogeriatric care in comparison to standard care for hip fracture patients in Germany within 1 year. We found the treatment in hospitals with collaborative orthogeriatric care to be more expensive, particularly due to inpatient and long-term care costs. The results stand in line with Prestmo et al. [27] who found increased acute admission costs due to collaborative orthogeriatric care. However, all other studies investigated in an existing literature review and meta-analysis [17] found reduced costs of collaborative orthogeriatric care during acute hospital admission, compared to the control group. Even the first mentioned study found nonsignificantly reduced costs in the collaborative orthogeriatric care group within 1-year follow-up [27].

Reasons might be different health and reimbursement systems in different countries, which restrict the comparability of the costs in these studies. Further reasons might be different study designs, different dealing with risk adjustment and different intensity and specific treatment of geriatric co-management, as there are different systems reaching from geriatric consultation to joint orthogeriatric ward [12]. Some studies investigated collaborative orthogeriatric care defined as consultations by geriatricians on request or on a regular basis, others focused on care, where the geriatrician is the primary attending physician who coordinates all services. Nevertheless, all studies investigated treatments which aimed at early discharge. All but one study [17] found a reduction of inpatient days which explains the reduced inpatient costs. However, the intervention we investigated comprises inpatient rehabilitation and does, therefore, not intend earlier hospital discharge than standard care.

For hip fractures, the German collaborative orthogeriatric care has an average hospital length of stay of approximately 20–25 days (without complications) [53] and includes early rehabilitation measures. It can only be reimbursed if the patient received collaborative orthogeriatric care for at least 14 days. The standard treatment without collaborative orthogeriatric care, however, lasts 11–12 days [53]. Thus, costs for collaborative orthogeriatric care are determined to increase. Particularly for standard care, but possibly also for collaborative orthogeriatric care, an additional subacute inpatient rehabilitation in a separate facility can be offered. As the rehabilitation for collaborative orthogeriatric care partly takes place already in the hospital, we summed up the inpatient length of stay due to the index hip fracture for the hospital and the rehabilitation and found on average 2.3 more inpatient days for patients treated in hospitals providing collaborative orthogeriatric care, which of course also increases costs.

A former study based on the same dataset and investigating the same intervention found a reduction of the mortality for patients treated in hospitals with collaborative orthogeriatric care [24]. This stands in line with the increased life years gained for patients in hospitals with collaborative orthogeriatric care in our study. Other studies found reduced mortality rates within 1 year as well [12, 17, 21, 25, 26]. Furthermore, we found an increase in time with an increased care dependence in terms of care level for the collaborative orthogeriatric care group. The increase in survival time may partly explain this: Patients who would have died may have survived after treatment in a hospital providing collaborative orthogeriatric care but with an increase in care dependence. Accordingly, we found an increase in long-term care costs for both the payer’s and the societal perspective.

The cost-effectiveness analysis revealed that the point estimate for the ICER was €52,378 per life year gained from a payer perspective and €62,419 per life year gained from a societal perspective, and €60,902 per QALY gained from a payer perspective and €81,614 per QALY gained from a societal perspective. For decision making, there is currently no generally accepted WTP threshold per life year or QALY gained. In the UK, a cost-effectiveness threshold ranging between ₤20,000 (€24,000) and ₤30,000 (€35,000) per QALY has been defined [54, 55], which is considerable lower than our results. For life-extending treatments at the end of life, this threshold was increased to ₤50,000 (€59,000) per QALY [56]. In the US, decision rules usually range between $50,000 (€45,000) and $100,000 (€91,000) per QALY [57], which would include this study’s ICER. The scientific basis and an update of this decision rules seem questionable and were discussed in literature [57–59]. As an example, it has been reported that oncology drugs usually exceed those thresholds and range between $100,000 (€91,000) and $150,000 (€136,000) per QALY [60]. In Germany, however, the WTP threshold is unknown.

To analyse the uncertainty in our results, we conducted an net benefit analysis and constructed cost-effectiveness acceptability curves which revealed that the intervention is cost-effective with great probability (> 95%) only for a very high WTP: From a payer perspective, collaborative orthogeriatric care can be considered cost-effective if the society is willing to pay at least €82,000 per life year gained, and from a societal perspective €95,000 per life year gained. The ICER per QALY is likely to be higher than the ICER per life-year gained, which suggests that the WTP per QALY would need to be even higher for a 95% probability of cost-effectiveness. Thus, when considering uncertainty, the required WTP may no longer remain within the formerly mentioned thresholds.

To the best of our knowledge, there are only two cost-effectiveness studies on collaborative orthogeriatric care until now. Prestmo et al. [27] calculated QALYs based on the EQ-5D-3L, and Ginsberg et al. [25] based on disability-adjusted life years. In both studies, collaborative orthogeriatric care was found to reduce costs and improve QALYs within 1 year. The first study conducted an uncertainty analysis. Collaborative orthogeriatric care had an 88% probability of being dominant, and a 99% probability of being cost-effective for a WTP of €62,500 per QALY [27]. Results regarding effectiveness stand in line with our results; differences due to costs were discussed above. However, these results clearly favour collaborative orthogeriatric care, whereas our study found very high ICER partly exceeding common threshold.

In our study, the high ICER may result from the fact that probably the effectiveness was underestimated and the costs were overestimated. We only considered 1-year follow-up due to data availability, but the time of survival are likely to increase when extending the follow-up. Costs were especially high in the beginning of the follow-up due to the high index hip fracture inpatient costs which, however, only occur once. With a longer follow-up, long-term care costs would of course increase, but total costs are likely to increase less sharply. Furthermore, the collaborative orthogeriatric care was implemented in German hospitals only recently. When increasing the follow-up or repeating the analysis using more recent data, learning effects of the hospitals can be expected.

Apart from that, our study has some limitations. First, the intervention and control group were defined on hospital level, not on patient level, which may not display effects correctly, because not all patients assigned in the intervention group actually received the treatment. However, when classifying intervention and control group on patient level we would have introduced a strong selection bias as only for patients who survived at least 14 days after surgery the collaborative orthogeriatric care procedure was recorded in the claims data. The share of patients in our data who actually were recorded as having received the collaborative orthogeriatric care procedure (OPS8-550) (and surviving at least 14 days) was 41% of all patients in the intervention group. However, other patients in the intervention group (i.e., having been treated in a hospital providing collaborative orthogeriatric care) may still have benefitted from the presence of a multidisciplinary geriatric team. Moreover, information on long-term care was only available on a quarterly period basis and no costs were available. We used information on care level and nursing home status to estimate the respective costs. However, costs for care level 0 from a societal perspective could not be estimated and costs from a payer perspective were used instead. In case of changing or newly occurring care level or nursing home status compared to the former quarterly period, it is unclear at what time within the quarterly period this happened. Also, short-term care could not adequately be considered. Therefore, the estimated costs may be rather rough. However, we applied a further societal perspective by calculating costs based on average wages for formal care and minimum daily care time per care level, which may underestimate the true long-term care costs. This perspective may be particularly informative for international comparisons. Furthermore, quality of life utilities were not observed in the patient population but estimated based on another study with a rather small population sample.

Despite these limitations, our study also has several important strengths. We used a large and rich data set, including information from almost 25,000 patients for the years 2012 through 2015. It was based on health and long-term care insurance claims data, which are less vulnerable to selection and information biases, an issue common for survey data. The health insurance has a high national coverage of about one third of the German population, which makes the results of our population-based study quite representative. We used entropy balancing, a reweighting algorithm to reduce confounding in observational studies due to potential selection bias and unbalanced baseline characteristics. There are studies which empirically demonstrated the superiority of EB in terms of least biased balancing weights compared to other balancing and matching methods, since it balances not only for means, but also for variance and skewness [44–46]. EB achieves higher covariate balance, does not discard individuals and obviates the need for manual propensity score model specification and balance checking, compared to propensity score matching. EB may work particularly well in large datasets like ours and for variables with frequent observations [45]. We addressed potential limitations of EB by ensuring consistent balancing constraints, by carefully checking the distributions of the covariates of the intervention and control group, and by excluding variables with too few observations. To our knowledge, this is the first study assessing costs of collaborative orthogeriatric care from a broad payer and societal perspective over a follow-up period of 12 months and with a large number of patients, both in Germany and worldwide.

## Conclusion

So far, this observational study is the largest economic evaluation of collaborative orthogeriatric care for geriatric hip fracture patients aged 80 years and older. Existing evidence suggests benefits when the treatment of older patients with hip fractures is organised as collaborative orthogeriatric care. This study supports this recommendation and shows that survival and quality-adjusted life years can be improved if patients are treated in hospitals providing collaborative orthogeriatric care, compared to hospitals offering treatment in traditional orthopaedic trauma wards. However, costs were found to increase due to collaborative orthogeriatric care, mainly driven by inpatient and long-term care costs. Therefore, collaborative orthogeriatric care can be considered cost-effective only if the society is willing to pay a certain amount of money per life year gained.

## Supplementary Information

Below is the link to the electronic supplementary material.

Supplementary file1 (DOCX 44 kb)

## Data Availability

The datasets supporting the conclusions of this article are owned by the German statutory health insurance AOK. Since public deposition of the data would breach ethical and legal compliance, data are only available upon formal request from the research institute of the AOK (WIdO). To request the data please contact the institutional body of the WIdO (wido@wido.bv.aok.de). To fulfill the legal requirements to obtain that kind of data, researchers must obtain a permission for a specific research question from the German Federal (Social) Insurance Office. Additionally, researchers must conclude a contract with the statutory health insurance regarding data access which can be requested from the "AOK-Bundesverband GbR" (Federal Association of Local Health Insurance Funds) under http://aok-bv.de/kontakt/. The licensee is permitted to use the data for the purpose of the research proposal within their company, exclusively. Thereby, company is defined as an economical unit. Licensees are not allowed to pass the data to a third party, or to create Software or data bases with the exception of scientific publications. Moreover, the study has to be approved by the data protection officer both at the statutory health insurance and the research institute. All calculations were performed using SAS software v9.4 (SAS Institute Inc, Cary, NC) and Stata 15 (StataCorp, College Station, TX).
