# Supplementary material for: Health-economic evaluation of collaborative orthogeriatric care for patients with a hip fracture in Germany: a retrospective cohort study using health and long-term care insurance claims data
Source: Eur J Health Econ. 2021 Apr 4;22(6):873–85. doi: 10.1007/s10198-021-01295-z (PMC8275532; doi:10.1007/s10198-021-01295-z)
Supplement: Supplementary file 1 — Supplementary file1 (DOCX 44 kb) [file 10198_2021_1295_MOESM1_ESM.docx]

**Health-economic evaluation of collaborative orthogeriatric care for patients with a hip fracture in Germany: A retrospective cohort study using health and long-term care insurance claims data**

European Journal of Health Economics

Claudia Schulz; Gisela Büchele; Raphael S. Peter; Dietrich Rothenbacher; Christian Brettschneider; Ulrich C. Liener; Clemens Becker; Kilian Rapp; Hans-Helmut König

Corresponding author:

Claudia Schulz, University Medical Center Hamburg-Eppendorf, Department of Health Economics and Health Services Research, Martinistr. 52, 20246 Hamburg, Germany, Phone +49 40 7410 54480, Fax +49 40 7410 40261, Email c.schulz@uke.de, ORCID: 0000-0003-1053-2126

## Supplementary Tables

Supplementary Table 1: Mean Quality of Life Utilities per Care Level

| Care level | N | Mean quality of life utilities ^a^ |
| --- | --- | --- |
| None | 555 | 0.8265 |
| 0 | 10 | 0.8193 |
| 1 | 194 | 0.6298 |
| 2 | 75 | 0.5085 |
| 3 | 22 | 0.4385 |
| ^a^ based on data on the EQ-5D-3L [1] from the AgeQualiDe-AgeCoDe study [2,3] and derived by the German tariff [4]. | | |

Supplementary Table 2: Mean, variance and skewness of the intervention and control group before and after entropy balancing

|  | OGCM group (N=14,005) | | | Control group (N=10,512) | | | | | |
| --- | --- | --- | --- | --- | --- | --- | --- | --- | --- |
|  |  |  |  | Before entropy balancing | | | After entropy balancing | | |
|  | Mean | Variance | Skewness | Mean | Variance | Skewness | Mean | Variance | Skewness |
| Male sex | 0.1994 | 0.1596 | 1.505 | 0.1978 | 0.1587 | 1.518 | 0.1993 | 0.1596 | 1.505 |
| Age at index hip fracture date [years] | 87.09 | 20.27 | 0.5238 | 87.17 | 20.99 | 0.5466 | 87.09 | 20.27 | 0.5235 |
| Care dependence during baseline [quarterly periods] |  |  |  |  |  |  |  |  |  |
| … in care level 0 | 0.1061 | 0.4683 | 7.765 | 0.1066 | 0.4479 | 7.759 | 0.1061 | 0.4683 | 7.764 |
| … in care level 1 | 2.48 | 10.31 | 0.8137 | 2.491 | 10.19 | 0.8036 | 2.48 | 10.31 | 0.8136 |
| … in care level 2 | 1.287 | 6.766 | 1.847 | 1.363 | 6.949 | 1.752 | 1.287 | 6.766 | 1.847 |
| … in care level 3 | 0.1704 | 1.004 | 6.633 | 0.2017 | 1.176 | 6.098 | 0.1704 | 1.005 | 6.631 |
| … living in a nursing home | 1.38 | 7.618 | 1.74 | 1.696 | 8.923 | 1.42 | 1.38 | 7.621 | 1.739 |
| Occurence of inpatient costs during baseline | 0.6526 | 0.2267 | -0.6411 | 0.6428 | 0.2296 | -0.596 | 0.6526 | 0.2267 | -0.6411 |
| Inpatient costs during baseline [€] | 6308 | 80900000 | 2.282 | 5660 | 69400000 | 2.481 | 6307 | 80900000 | 2.282 |
| Outpatient costs during baseline [€] | 1700 | 1413582 | 2.398 | 1767 | 1436238 | 2.239 | 1700 | 1413687 | 2.398 |
| Medication costs during baseline [€] | 2327 | 5613924 | 2.328 | 2335 | 5602612 | 2.311 | 2327 | 5613808 | 2.328 |
| Devices/medical appliances costs during baseline [€] | 244.5 | 425982 | 4.764 | 259.3 | 462341 | 4.556 | 244.5 | 426082 | 4.763 |
| Index hospital volume (annual hip fracture cases) | 89.4 | 1393 | 0.4445 | 72.05 | 1197 | 1.041 | 89.35 | 1394 | 0.4447 |
| Medication-based comorbidities |  |  |  |  |  |  |  |  |  |
| … Acid related disorders | 0.5165 | 0.2497 | -0.06587 | 0.522 | 0.2495 | -0.08798 | 0.5165 | 0.2498 | -0.06594 |
| … Bone diseases (osteoporosis) | 0.09411 | 0.08526 | 2.78 | 0.08866 | 0.08081 | 2.894 | 0.09411 | 0.08526 | 2.78 |
| … Cancer | 0.005569 | 0.005539 | 13.29 | 0.006754 | 0.006709 | 12.04 | 0.005572 | 0.005541 | 13.28 |
| … Cardiovascular diseases (incl. hypertension) | 0.8743 | 0.1099 | -2.259 | 0.8788 | 0.1065 | -2.321 | 0.8743 | 0.1099 | -2.259 |
| … Dementia | 0.09925 | 0.08941 | 2.681 | 0.11 | 0.09789 | 2.493 | 0.09927 | 0.08943 | 2.68 |
| … Diabetes mellitus | 0.2126 | 0.1674 | 1.405 | 0.2101 | 0.166 | 1.423 | 0.2126 | 0.1674 | 1.405 |
| … Epilepsy | 0.09954 | 0.08963 | 2.675 | 0.09675 | 0.08739 | 2.728 | 0.09953 | 0.08963 | 2.675 |
| … Glaucoma | 0.1002 | 0.09021 | 2.662 | 0.09494 | 0.08593 | 2.764 | 0.1002 | 0.0902 | 2.662 |
| … Gout, Hyperuricemia | 0.1371 | 0.1183 | 2.11 | 0.1335 | 0.1157 | 2.156 | 0.1371 | 0.1183 | 2.11 |
| … HIV |  |  |  |  |  |  |  |  |  |
| … Hyperlipidemia | 0.2835 | 0.2032 | 0.9605 | 0.2794 | 0.2014 | 0.9833 | 0.2835 | 0.2032 | 0.9606 |
| … Intestinal inflammatory diseases | 0.009282 | 0.009197 | 10.23 | 0.01142 | 0.01129 | 9.198 | 0.009289 | 0.009203 | 10.23 |
| … Iron deficiency anemia | 0.0824 | 0.07561 | 3.037 | 0.08533 | 0.07806 | 2.969 | 0.0824 | 0.07562 | 3.037 |
| … Migraines |  |  |  |  |  |  |  |  |  |
| … Pain | 0.605 | 0.239 | -0.4296 | 0.5965 | 0.2407 | -0.3932 | 0.605 | 0.239 | -0.4296 |
| … Parkinson’s disease | 0.0744 | 0.06887 | 3.244 | 0.07354 | 0.06813 | 3.268 | 0.0744 | 0.06887 | 3.244 |
| … Psycholgical disorders (sleep disorder, depression) | 0.3846 | 0.2367 | 0.4742 | 0.3903 | 0.238 | 0.4497 | 0.3847 | 0.2367 | 0.4741 |
| … Psychoses | 0.2311 | 0.1777 | 1.276 | 0.2595 | 0.1922 | 1.097 | 0.2311 | 0.1777 | 1.276 |
| … Respiratory illness (asthma, COPD) | 0.1499 | 0.1274 | 1.962 | 0.1503 | 0.1277 | 1.957 | 0.1499 | 0.1274 | 1.962 |
| … Rheumatologic conditions | 0.4381 | 0.2462 | 0.2494 | 0.4331 | 0.2456 | 0.2699 | 0.4381 | 0.2462 | 0.2494 |
| … Thyroid disorders | 0.2146 | 0.1686 | 1.39 | 0.2148 | 0.1687 | 1.389 | 0.2146 | 0.1686 | 1.39 |
| … Tuberculosis |  |  |  |  |  |  |  |  |  |

OGCM = Orthogeriatric co-management

Supplementary Table 3: Estimated Time per Care Level and Nursing Home Status During Follow-up

| Time within follow-up | OGCM group | Control group | Difference | (SE) |
| --- | --- | --- | --- | --- |
| … without care level [quarterly periods] ^a^ | 0.80 | 0.81 | -0.01 | (0.02) |
| … in care level 0 [quarterly periods] ^a^ | 0.04 | 0.03 | 0.01 | (0.00) |
| … in care level 1 [quarterly periods] ^a^ | 1.07 | 0.96 | 0.11*** | (0.02) |
| … in care level 2 [quarterly periods] ^a^ | 1.03 | 1.00 | 0.03 | (0.02) |
| … in care level 3 [quarterly periods] ^a^ | 0.33 | 0.36 | -0.03 | (0.01) |
| … with an increased care level [quarterly periods] ^a, b^ | 1.36 | 1.34 | 0.02 | (0.02) |
| … lost due to death [days/quarterly periods] ^c^ | 85.10 / 0.95 | 93.34 / 1.04 | -8.24 / -0.09*** | (1.97 / 0.01) |
| … in nursing home [quarterly periods] ^a^ |  |  |  |  |
| - for all patients | 1.22 | 1.21 | 0.01 | (0.02) |
| - for patients not living in nursing home at baseline (N=18,059) | 0.78 | 0.75 | 0.03 | (0.02) |
| OGCM = Orthogeriatric co-management. *p<.05; **p<.01; ***p<.001. SE = Standard error. ^a^ Estimated using a weighted Poisson regression. ^b^ Compared to care level in quarterly period before index fracture. ^c^ Mean weighted deceased time within follow-up. Note that the time without care level, in care level 0-3 and deceased sums up to more than 4 quarterly periods because death was measured on a daily basis and care level on a quarterly period basis. | | | | |

1. EuroQol Group: EuroQol-a new facility for the measurement of health-related quality of life. Health policy (Amsterdam, Netherlands) **16**(3), 199-208 (1990).

2. Luck, T., Riedel-Heller, S.G., Kaduszkiewicz, H., Bickel, H., Jessen, F., Pentzek, M., Wiese, B., Koelsch, H., van den Bussche, H., Abholz, H.-H.: Mild cognitive impairment in general practice: age-specific prevalence and correlate results from the German study on ageing, cognition and dementia in primary care patients (AgeCoDe). Dementia and geriatric cognitive disorders **24**(4), 307-316 (2007). doi:10.1159/000108099

3. König, H.-H., Brettschneider, C., Lühmann, D., Kaduszkiewicz, H., Oey, A., Wiese, B., Werle, J., Weyerer, S., Fuchs, A., Pentzek, M.: EQ-5D-3L health status and health state utilities of the oldest-old (85+) in Germany: results from the AgeCoDe-AgeQualiDe study. Quality of Life Research **29**(12), 3223-3232 (2020). doi:10.1007/s11136-020-02597-0

4. Greiner, W., Claes, C., Busschbach, J., von der Schulenburg, J.-M.G.: Validating the EQ-5D with time trade off for the German population. The European journal of health economics **6**(2), 124-130 (2005). doi:10.1007/s10198-004-0264-z
